# Clonal Spread of *Mycoplasma pneumoniae* in Primary School, Bordeaux, France

**DOI:** 10.3201/eid1802.111379

**Published:** 2012-02

**Authors:** Sabine Pereyre, Hélène Renaudin, Alain Charron, Cécile Bébéar

**Affiliations:** Université de Bordeaux, Bordeaux, France; Institut National de la Recherche Agronomique, Bordeaux;; Centre Hospitalier Universitaire de Bordeaux, Bordeaux

**Keywords:** Mycoplasma pneumoniae, bacteria, bacterial typing techniques, disease outbreaks, primary school, France

**To the Editor**: *Mycoplasma pneumoniae* is responsible for ≈20% of all cases of community-acquired pneumonia. The most common form of the infection is tracheobronchitis, for which an etiologic diagnosis is seldom reached (*1*). Although tracheobronchitis is often mild, the infection is disruptive, with the cough lasting several weeks, and consumes substantial resources (*2*). *M. pneumoniae* infections occur endemically and epidemically worldwide, especially in children and young adults (*1*). In 2010, an increased incidence was reported from Denmark (*3*), England and Wales (*4*), and Israel (*5*). Several outbreaks have been reported in closed or semiclosed settings, as indicated on the basis of similar clinical symptoms, chest radiograph results, and detection of the bacteria (*1*).

Previous *M. pneumoniae* typing methods were based on the analysis of the gene encoding the cytadhesin P1 (MPN141) or the gene *MPN528a* (*6*). These methods only enabled the separation of isolates into 2 types and a few variants; therefore, clinical isolates were previously poorly differentiated. We recently developed a multilocus variable-number tandem repeat analysis (MLVA), based on the study of the whole genome, that can differentiate >26 distinct variable-number tandem repeat types (*7*). We report the use of this MLVA typing method to show evidence of a clonal spread of a unique strain of *M. pneumoniae* among children in a French primary school and their household contacts.

In January 2011, 6 children (4–9 years of age), who attended the same primary public school in Bordeaux, France, reported fever, pharyngitis, rhinorrhea, and dry cough that later became mucoid. One of the children was admitted to the pediatric ward of the University Hospital of Bordeaux, and atypical pneumonia was confirmed by radiologic testing. A diagnosis of tracheobronchitis was confirmed by general practitioners for the 5 other children. Three of the children were administered β-lactam antimicrobial drugs that did not modify the course of the illness. An additional child (4 years of age), a first cousin of one of the 6 case-patients, also received a diagnosis of tracheobronchitis after repeated contact with his cousin.

Throat swab or blood samples were obtained from the 7 children, and throat swab samples were obtained from the household members of 4 of their families. DNA was extracted from throat specimens, and a TaqMan real-time PCR was performed to detect *M. pneumoniae* as described (*8*). MLVA typing was performed on the same DNA extracts, according to the method of Dégrange et al. (*7*). *M. pneumoniae*–specific IgM and IgG in serum specimens were assessed by ELISA. PCR was used to detect *Bordetella pertussis*, *B. parapertussis*, *Chlamydia pneumoniae*, *Streptococcus pneumoniae*, and viruses commonly responsible for respiratory tract infections. In France, 10% of *M. pneumoniae* isolates are resistant to macrolides (*9*); thus, we used real-time PCR and melting curve analysis to detect macrolide resistance–associated mutations in the 23S rRNA gene (*9*).

The 7 children were confirmed to be positive for *M. pneumoniae* infection by PCR or by the presence of *M. pneumoniae–*specific IgM (Figure). No other respiratory tract pathogens were found. In all cases, MLVA determined the strain type to be 34572, also called MLVA type J (*7*); this finding suggests clonal spread of a specific *M. pneumoniae* strain. No macrolide resistance*–*associated mutation was found in the 23S rRNA gene. All children were treated with roxithromycin or clarithromycin and rapidly recovered, although PCR results remained positive for up to 6 weeks in subsequent throat samples. This length of persistence is in accordance with a previous study showing that the median time for carriage of *M. pneumoniae* DNA was 7 weeks after disease onset and that adequate treatment did not shorten this period (*10*).

**Figure:**
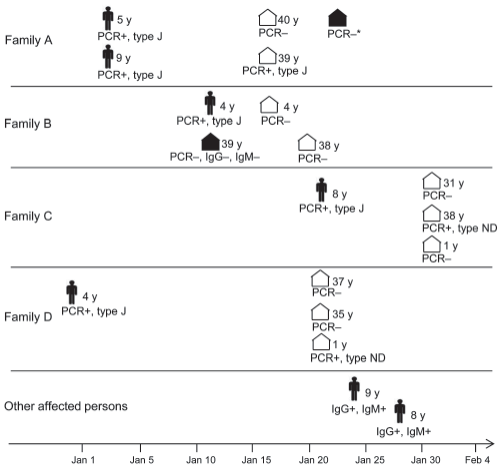
Timing and characteristics of patients and contacts in a study using the multilocus variable-number tandem repeat (MLVA) typing method to show evidence of clonal spread of a unique strain of *Mycoplasma pneumoniae* among children attending a French primary school and their household contacts. Dates correspond to the date of specimen collection during December 30, 2010–February 1, 2011. Figure shapes indicate affected children, by age in years; white house shapes indicate asymptomatic household contacts; black house shapes indicate household contacts with respiratory symptoms. PCR+, throat swab specimen positive by *M. pneumoniae*–specific real-time PCR; PCR, throat swab specimen negative by *M. pneumoniae*–specific real-time PCR; type J, MLVA type J; type ND, MLVA type not determined; *PCR performed after an 8-day macrolide treatment; IgG+, IgM+, presence of specific *M. pneumoniae* IgG and IgM in serum; IgG–, IgM–, absence of specific *M. pneumoniae* IgG or IgM, respectively, in serum.

*M. pneumoniae* DNA was also found in throat swab specimens of 3 household contacts (2 adults and a 1-year-old child) in 3 separate families (Figure). The MLVA type was determined in 1 contact; it also was MLVA type J, suggesting that carriage in this contact was related to spread of the same clone. Of interest, none of these 3 household members had respiratory symptoms. Nilsson et al. (*10*) also reported a high frequency of *M. pneumoniae* DNA carriage in household contacts; however, in contrast to contacts in our study, all of the household contacts in the study by Nilsson et al. had ongoing or recent respiratory tract symptoms.

In summary, we report an outbreak of *M. pneumoniae* infections confirmed by MLVA, a discriminatory typing method. MLVA typing revealed the clonal spread of a single *M. pneumoniae* type J strain in children attending the same primary school and in their household contacts. The cases we identified may represent only a small proportion of the actual cases, which were likely underestimated due to mild symptoms, poor knowledge of *M. pneumoniae* infections by general practitioners, and lack of PCR availability. We showed that MLVA typing of *M. pneumoniae* can be used to detect clonal spread and outbreaks. This approach might also be useful for studying the worldwide emergence of *M. pneumoniae* macrolide resistance and for finding resistant clones with the potential for spreading.
